# Association between polymorphisms in the *SOX9* region and canine disorder of sex development (78,XX; *SRY*-negative) revisited in a multibreed case-control study

**DOI:** 10.1371/journal.pone.0218565

**Published:** 2019-06-20

**Authors:** Joanna Nowacka-Woszuk, Izabela Szczerbal, Monika Stachowiak, Maciej Szydlowski, Wojciech Nizanski, Stanislaw Dzimira, Artur Maslak, Rita Payan-Carreira, Eline Wydooghe, Tomasz Nowak, Marek Switonski

**Affiliations:** 1 Department of Genetics and Animal Breeding, Poznan University of Life Sciences, Poznan, Poland; 2 Department of Reproduction and Clinic of Farm Animals, Wroclaw University of Environmental and Life Sciences, Wroclaw, Poland; 3 Department of Pathology, Wroclaw University of Environmental and Life Sciences, Wroclaw, Poland; 4 Vital-Vet Veterinary Clinic, Pniewy, Poland; 5 CECAV, Centro de Ciência Animal e Veterinária, Universidade de Trás-os-Montes e Alto Douro, Quinta de Prados, Vila Real, Portugal; 6 Department of Reproduction, Obstetrics, and Herd Health, Clinic of Small Animal Reproduction, Ghent University, Merelbeke, Belgium; 7 Department of Animal Reproduction, Poznan University of Life Sciences, Poznan, Poland; Leibniz Institute on aging - Fritz Lipmann Institute (FLI), GERMANY

## Abstract

Testicular or ovotesticular disorders of sex development (DSD) in individuals with female karyotype (XX) lacking the *SRY* gene has been observed in several mammalian species, including dogs. A genetic background for this abnormality has been extensively sought, and the region harboring the *SOX9* gene has often been considered key in canine DSD. Three types of polymorphism have been studied in this region to date: a) copy number variation (CNV) in a region about 400 kb upstream of *SOX9*, named CNVR1; b) duplication of *SOX9*; and c) insertion of a single G-nucleotide (rs852549625) approximately 2.2 Mb upstream of *SOX9*. The aim of this study was thus to comprehensively analyze these polymorphisms in a large multibreed case-control cohort containing 45 XX DSD dogs, representing 23 breeds. The control set contained 57 fertile females. Droplet digital PCR (ddPCR) was used to study CNVR1 and the duplication of *SOX9*. Fluorescent *in situ* hybridization (FISH) was used to visualize copy numbers on a cellular level. The Sanger sequencing approach was performed to analyze the region harboring the G-insertion. We confirmed that CNVR1 is highly polymorphic and that copy numbers varied between 0 and 7 in the case and control cohorts. Interestingly, the number of copies was significantly higher (P = 0.038) in XX DSD dogs (mean = 2.7) than in the control females (mean = 2.0) but not in all studied breeds. Duplication of the *SOX9* gene was noted only in a single XX DSD dog (an American Bully), which had three copies of *SOX9*. Distribution of the G-nucleotide insertion was similar in the XX DSD (frequency 0.20) and control (frequency 0.14) cohorts. Concluding, our study showed that CNVR1, located upstream of *SOX9*, is associated with the XX DSD phenotype, though in a breed-specific manner. Duplication of the *SOX9* gene is a rare cause of this disorder in dogs. Moreover, we did not observe any association of G-insertion with the DSD phenotype. We assume that the genetic background of XX DSD can be different in certain breeds.

## Introduction

Development of a testis or ovotestis in mammals with a female set of sex chromosomes (XX) and lacking the *SRY* gene has been described in several species, including humans [1] and domestic animals such as goats, pigs, horses, and dogs [2]. Extensive studies of the molecular background of this disorder have been carried out in humans, and various causative DNA variants—including point mutations of *RSPO1* or *WNT4*, duplication of *SOX3* or *SOX9*, deletion in *RSPO1* and copy number variation (CNV) of regulatory sequences located upstream of *SOX9*—have been found [1]. Among these, an increased number of copies in CNV appeared to be the most common feature observed in XX DSD patients. The *SOX9* region has thus been extensively studied in XX DSD dogs [3–8], pigs [9, 10], and a cat [11].

The *SOX9* gene, whose expression is upregulated by SRY transcription factor in the fetal testis, plays a crucial role in the sexual differentiation of males. The first study of canine *SOX9* showed that its quantity and temporal pattern of expression in normal gonads is similar to that in other mammals [12]. Attempts to find *SOX9* polymorphism in XX DSD dogs showed that the identified DNA variants were not associated with this abnormality [13, 14]. On the other hand, Rossi et al [4] reported that some XX DSD dogs carry a duplicate of *SOX9*, and Marcinkowska-Swojak et al [5] found a CNV upstream of *SOX9* (named CNVR1) in both DSD and control dogs. Deep sequencing of this region showed that it contains a fragment homologous to the *MAGI2* gene consisting of its 5’-flanking regulatory sequences. It is known that a normal *MAGI2* is located on canine chromosome 18 (CFA18), while *SOX9* resides on CFA9 [7]. Finally, Meyers-Wallen et al [8] postulated that a single nucleotide G-insertion (rs852549625), located ~2.2 Mbp upstream of *SOX9*, is a marker associated with canine XX DSD.

These studies of the canine *SOX9* region have shown that the importance of these polymorphisms needs further elucidation. The goal of our study was thus to comprehensively analyze the polymorphisms in this region, including CNVR1 and single nucleotide G-insertion in the 5’-flanking region of *SOX9*, as well as duplication of *SOX9*, in a multibreed cohort of 45 XX DSD dogs and 57 control XX dogs.

## Material and methods

### Animals

The cohort of 45 XX DSD cases representing 23 breeds was included in this study. A control group of 57 fertile, unrelated females was composed from animals of the same breeds as the cases. Details concerning breed and gonadal status, as well as the numbers of controls, are shown in Table A in S1 File. In the case of four DSD animals, we also collected blood samples from their relatives: family A (of a French Bulldog), family B (of a Pug) and family C (of a Cane Corso) (Table B and Figure A in S1 File). Two generations of family B have previously been described by Marcinkowska-Swojak et al [5]; here it was possible to include the grandparents, allowing us to study the three-generational segregation of the polymorphisms. The blood samples (3 ml) were collected with the consent of the owners, after receiving approval from the local Bioethical Commission for Animal Care and Use in Poznan, Poland, in line with standard Polish veterinary protocols.

### Genetic diagnosis of DSD cases

New DSD cases were diagnosed with the use of cytogenetic, molecular and histological techniques. Chromosome preparations were obtained from *in vitro* cultured lymphocytes. Sex chromosomes were identified based on their bi-armed morphology on Giemsa stained slides. For each case at least 50 metaphase spreads were analyzed. PCR detection of *SRY* gene was performed with the use of primers described earlier by Switonski et al [15]. Tissue sections obtained from gonads fixed with 10% formalin were stained following the Masson–Goldner method (haematoxylin and eosin).

### Droplet-digital PCR for CNV analysis

The CNV polymorphisms were studied using the droplet-digital PCR (ddPCR) method. Five assays were designed to amplify and analyze CNVR1 and the *SOX9* gene, including its exon 1, exon 2, and exon 3 as well as 5’-flanking region (13.5 kb upstream of *SOX9*). The *HSD17B7* gene was used as a reference gene. Details of the primers and probe sequences, as well as their location in the canine genome, are given in Table C in S1 File. The procedure was performed in line with the manufacturer’s protocol, as described earlier by Szczerbal et al [16]. Briefly, the PCR mixture was composed of ddPCR Supermix for Probes (Bio-Rad), fluorescence labeled probes, primers, a restriction enzyme (*Hae*III or *Hind*III), DNA, and water. The mixture was spliced into oil droplets and the emulsion PCR was run. Next the fluorescence in the droplets was detected using a QX200 droplet reader (Bio-Rad). The results were analyzed using Quantasoft software (Bio-Rad).

### Fluorescence *in situ* hybridization (FISH)

The FISH technique was used to visualize the CNVR1 in a studied families. Cytogenetic preparations were obtained from a short-term lymphocyte culture. Two BAC probes obtained from CHORI-82 Canine Boxer (F) (*Canis familiaris*) BAC library (https://bacpac.chori.org/) were used. The CH82-116C01 clone spans the *SOX9* gene and was labeled by random priming with digoxigenin-11-dUTP, and detected using anti-digoxigenin-fluorescein (green signals). The CH82-26L13 clone used to identify CNVR1, was labeled with biotin-16-dUTP and detected using streptavidin-Cy3 (red signals). Hybridization was performed following a procedure described by Marcinkowska-Swojak et al [5]. Slides were examined under a Nikon E 600 Eclipse fluorescence microscope equipped with a cooled digital CCD camera and driven by LUCIA computer-aided software.

### DNA Sanger sequencing

DNA was isolated with MasterPure DNA Purification Kit for Blood Version II (Epicentre, Illumina) from peripheral blood lymphocytes. The quantity was checked on a Qubit 2.0 fluorometer (Invitrogen). The studied fragment overlapped with the single nucleotide insertion (G-insertion; rs852549625) located upstream of the *SOX9* gene (position on CFA9: 6048201_6048202), as indicated by Meyers-Wallen et al [8]. The amplicon of 839 bp was obtained after the conventional PCR reaction with the use of the following primers: F: 5’ GTCCTGACCTTGGTGGGTCT and R 5’ GAGGCCCTAGAAAAGGATGG. The PCR products were checked on the 1.5% agarose gel, and then underwent enzymatic purification with exonuclease I and alkaline phosphatase (Thermo Fisher Scientific). Next, amplification with BigDye Terminator v3.1 Cycle Sequencing Kit (Thermo Fisher Scientific) was conducted and the amplicons were purified on the filtration plate using Sephadex G50 (Sigma). Capillary electrophoresis was run on a Genetic Analyzer 3130 (Applied Biosystems) and the results were analyzed using the DNAStar software package.

### Statistical analysis

A nonparametric Wilcox test was performed to assess whether the distribution of CNV differed between the DSD and control groups. The haplotypes were reconstructed using the hap procedure (R software environment, ‘gap’ package version 1.1–22). To test associations between individual haplotypes and DSD, we calculated the exposure odds ratio with its 95% confidence interval and determined whether the value 1.0 was excluded from the confidence interval.

## Results

In this study 45 XX DSD dogs, including 26 new ones, were included (Table A in S1 File). All new cases had enlarged clitoris, a normal set of female sex chromosomes (78,XX) and lack of *SRY* gene (Figure B in S1 File.). For 20 dogs histological analysis of the gonads was performed and the following results were obtained: two testes (n = 10, Figure B in S1 File), one testis and one ovotestis (n = 1) and two ovotestes (n = 8) and one testis and one ovary with tumor (n = 1) (Table A in S1 File).

### Duplication of *SOX9* is rare in XX DSD dogs

**Fig 1:**
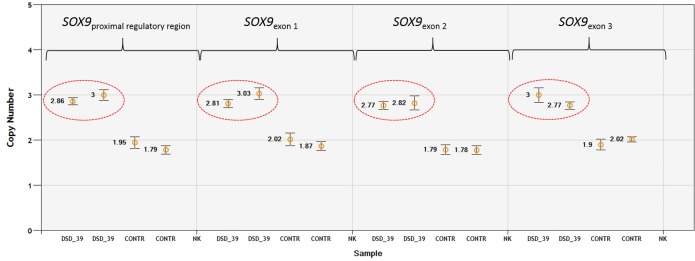
Identification of 3 copies of *SOX9* gene in a single DSD–39 case by ddPCR. Following fragments were analyzed: proximal regulatory region, 13.5kb upstream; exon 1; exon 2; exon 3. Three copies for the studied DSD case (analyzed in duplicate) have been marked in a red ellipse; CONTR: control animals with two copies of *SOX9*; NK: negative control (no DNA). Error bars represent the 95% confidence interval.

**Fig 2:**
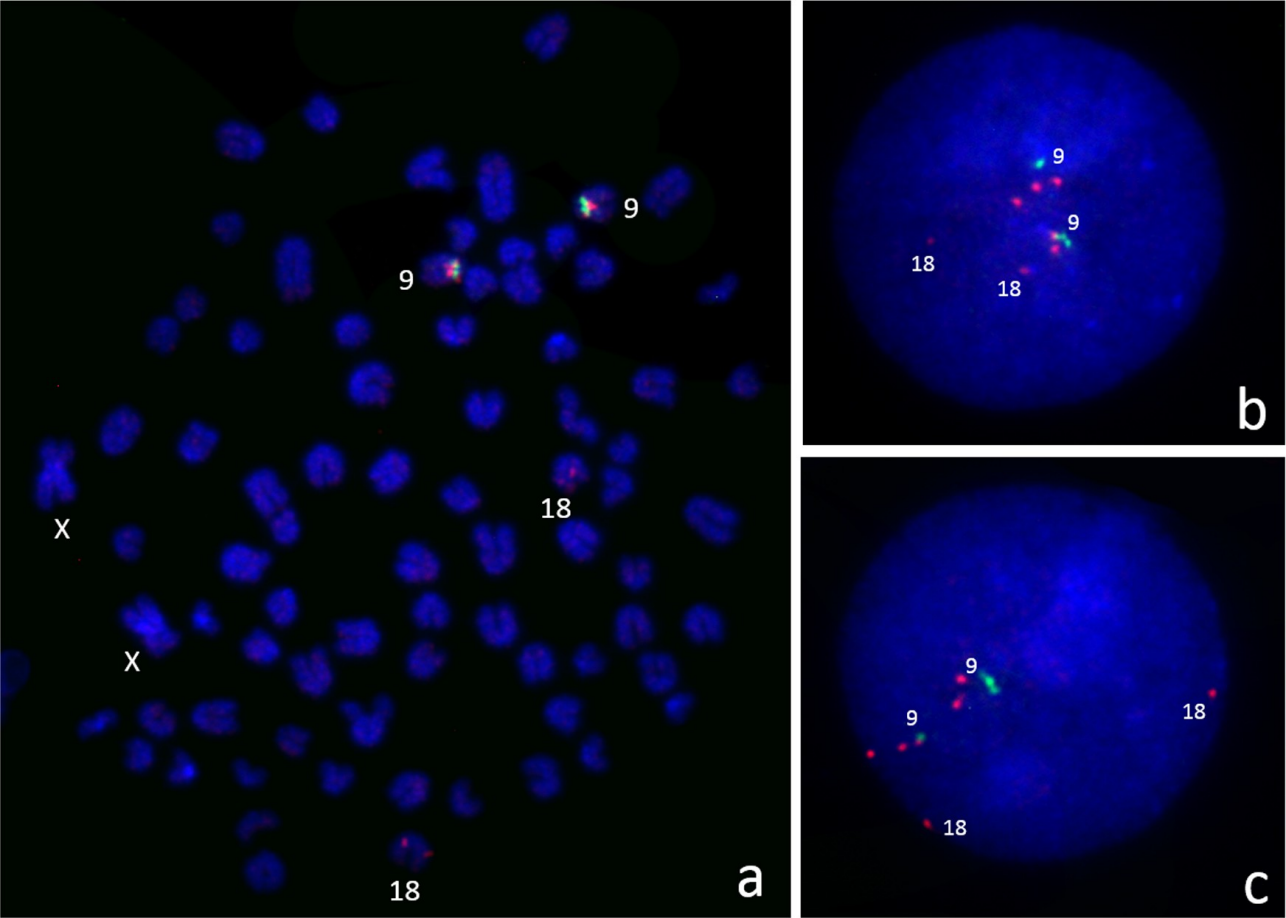
Visualization of copy numbers of *SOX9* gene (green) and CNVR1 (red) in the DSD-39. (a) Mapping of FISH probes on metaphase spread. (b–c) Examples of FISH on interphase nuclei: three copies of the *SOX9* gene and multiple copies of CNVR1 were detected. Note that the BAC probe specific to CNVR1 also hybridized to CFA18 due to a homologous fragment.

To analyze copy number variation, two genomic regions from CFA9 were selected: the entire *SOX9* gene and the CNV upstream of *SOX9* (CNVR1). The number of copies of the *SOX9* gene was estimated in 45 DSD cases and in 57 control dogs using an assay for exon 2. Two copies of this gene were found (Table D in S1 File) in all but one case, DSD-39 (an American Bully), had three copies of the *SOX9* gene (Fig 1). To verify whether the entire coding region and its proximal regulatory sequences were duplicated, three additional assays covering exon 1, exon 3, and the 5’-flanking region located 13.5 kb upstream of *SOX9* were designed. These confirmed that the duplication spans the entire studied region. Moreover, the FISH analysis of the interphase nuclei from DSD-39 allowed use to visualize the presence of the extra copy of the *SOX9* gene (Fig 2).

### CNVR1 upstream of *SOX9* and its association with XX DSD phenotype

The analysis of CNVR1 showed high variability in the copy number. In both the DSD and control groups, the number of copies varied from 0 to 7 (Figure C and Table D in S1 File). The distribution of this polymorphism showed significant association with DSD phenotype (P = 0.038) when all cases were included in the analysis. The mean copy number was 2.7 in the DSD group and 2.0 in the controls. To verify the breed-specific association of this polymorphism with the DSD phenotype, a distribution analysis was performed for the two most represented breeds in DSD cohort (French Bulldog and American Staffordshire Terrier); however, no statistical significance was found. In the next step, the analysis was performed again, but excluding these two breeds. The difference in mean copy number remained significant (P = 0.045 with both breeds excluded, P = 0.048 with French Bulldogs excluded, and P = 0.035 with American Staffordshire Terriers excluded; Table 1). The segregation of CNVR1 in families was most informative for family B (Pug), in which the two DSD cases (DSD-27 and DSD-28) had seven and six copies, respectively (Table D in S1 File). The results indicate that the increased number of CNVR1 may by associated with DSD phenotype, but this association seems to be breed-specific.

**Table 1:** Copy number variation in the DSD and control groups.

Groups	CNVR1	
	mean copy number	P value
DSD all cases (n = 44)*	2.7	0.038
control all (n = 57)	2.0	
DSD French Bulldog (n = 12)	2.2	0.699
control French Bulldog (n = 8)	1.6	
DSD without French Bulldog (n = 32)	2.9	0.048
control without French Bulldog (n = 49)	2.1	
DSD American Staffordshire Terrier (n = 6)	3.0	1.000
control American Staffordshire Terrier (n = 4)	2.5	
DSD without American Staffordshire Terrier (n = 38)	2.6	0.035
control without American Staffordshire Terrier (n = 53)	2.0	
DSD without French Bulldog and American Staffordshire Terrier (n = 26)	2.8	0.045
control without French Bulldog and American Staffordshire Terrier (n = 45)	2.0	

* DSD-39 was excluded from this analysis, as it was a carrier of *SOX9* gene duplication

### Lack of association between G-insertion (rs852549625) and DSD phenotype

The distribution of G-insertion (rs852549625) was determined in 44 DSD cases and 57 controls (Table D in S1 File). The ins/ins genotype was very rare in both groups, similarly to the heterozygous status (del/ins), while the del/del genotype was predominant (Table E in S1 File). We also examined the segregation of G-insertion in the three studied families. All members of family A (French Bulldog) were del/del homozygotes. In the three-generation Pug family (B), the ins/ins genotype was observed in two affected animals and in their father (Figure D in S1 File). On the other hand, the ins/ins genotype was observed in the Cane Corso family (C) only for the healthy mother of the DSD case, whereas the DSD case and its healthy sister were heterozygotes (del/ins) (Table D in S1 File). These results indicate that G-insertion is not related to DSD phenotype in our cohorts.

Additional analysis was performed to verify whether G-insertion cosegregates with the patterns of CNVR1 detected using the FISH technique. Interestingly, in the Pug family (B) all members displayed complete accordance between both polymorphisms: the del/del genotype was observed only for members with no copies of CNVR1 (no FISH signal on both CFA9); a FISH signal was detected only on one CFA9 in the animals with del/ins genotype; the animals with the ins/ins genotype had multiplication of CNV on both CFA9 (Figure D in S1 File). Similar accordance between G-insertion genotype and FISH signal patterns was observed for Cane Corso family (C) (Figure E in S1 File) On the other hand, there was no compatibility between the two genotypes in the French Bulldog trios (family A), since the mother of the DSD case had del/del genotype for G-insertion, but was also a carrier of CNV on one copy of CFA9 (Figure F in S1 File).

We additionally checked the frequencies of other variants identified in the examined PCR amplicon. Altogether, we found 4 SNPs (rs22666734, rs9120764, rs851784236, rs852192717; see Table D in S1 File), which together with G-insertion (rs852549625) cosegregated as five haplotypes. Analysis of the haplotype frequencies did not show any association with DSD phenotype (Table F in S1 File).

## Discussion

Testicular or ovotesticular XX DSD, is the most common form of DSD other than cryptorchidism in dogs, and has been diagnosed in dozens of breeds. However, its incidence has not been estimated to date. Meyers-Wallen [17] published a list of 29 breeds in which this disorder has been reported. In the following years, cases representing ten additional breeds were reported (Table G in S1 File), including six from this study. We here examined 45 DSD dogs representing 23 breeds, among which the best represented were French Bulldogs (n = 12) and American Staffordshire Terriers (n = 6). In a similar study of 64 XX DSD dogs, reported by Meyers-Wallen et al [8], affected dogs were predominantly of the American Cocker Spaniel, English Cocker Spaniel, and Kerry Blue Terrier breeds. This information shows that this disorder is a serious health problem in many breeds, and determining its molecular background is important for dog breeders and veterinarians.

The mode of inheritance of this disorder in dogs is still not clear. Initially, a sex-dependent monogenic, autosomal recessive inheritance model was proposed [18], but identification of the associated CNV suggested monogenic, autosomal, and dominant inheritance [5]. Recently, a multifactorial background with breed-dependent penetrance has been suggested by Meyers-Wallen et al [8]. In this study, we analyzed a three-generation Pug family in which two XX DSD cases had seven and six copies of CNVR1 and ins/ins genotype at the G-insertion site. Segregation analysis of the polymorphic variants suggests that the causative mutation associated with these polymorphisms was inherited from a father with ins/ins genotype and five copies of CNVR1, suggesting a sex-dependent model of inheritance. On the contrary, in the Cane Corso family, both the XX DSD case and her healthy sister had the same heterozygous genotype (del/ins), while their healthy mother was a homozygote (ins/ins). All animals had low copy numbers at CNVR1. We therefore suppose that canine XX DSD is not caused by the same mutation in affected breeds.

PCR-based methods, such as ddPCR, facilitate estimating the total number of copies in CNV regions, but do not allow the number of repeats on homologous chromosomes to be distinguished [19]. It is a challenging task to analyze the multiallelic CNVs (mCNVs) that are known to play a role in the human gene–dose variation that results in many human diseases [20, 21]. The CNVR1 studied here is an example of an mCNV, as up to seven copies were observed. The FISH technique applied for some of the DSD cases helped determine here how the copies of CNVR1 are located on each CFA9. We hypothesize that the breed-specific effect of CNVR1 may depend on its distribution on homologous chromosomes, and that multiplication on a single chromosome is sufficient to induce a locus–dosage effect.

This study performed on larger cohort confirmed our earlier suggestion concerning occurrence of higher number of copies of CNVR1 in XX DSD dogs [5, 6]. However, this observation was not universal for all DSD cases, which indicate a breed-specific relationship between this type of polymorphism and DSD phenotype. We can speculate that this region might have functional relevance and may triggers expression of *SOX9* in the absence of *SRY*. In humans, duplications upstream of *SOX9* lead to the development of an XX sex reversal phenotype (for review see: Croft et al [22]). It has been shown that duplication of the *SOX9* locus affects the organization of topologically associated domains (TADs), which are thought to direct regulatory elements to promoters of target genes [23, 24]. Moreover, chromatin folding is important in the regulation of cell type-specific *SOX9* expression [25]. Recently, a detailed analysis of the upstream regulatory landscape of human *SOX9* helped identify three putative enhancers [26] that respond to testis-specific regulators, including SRY, SF1, and SOX9 itself; thus mutations in these sequences may therefore lead to DSD phenotype. Unfortunately, there has been no information to date on canine enhancers of *SOX9*. It seems that these regulatory elements may act in a species-specific manner. For example, Testis Specific Enhancer (TES) with TESCO core (located 13.5 kb upstream) is a well-known enhancer of *Sox9* in mice [27, 28], though in humans orthologous sequences do not play a role in *SOX9* regulation [26]. This suggestion is also supported by the fact that no human patients have been diagnosed with pathogenic variants in the TESCO sequence [29]. In our study, we also identified only a single case in 45 (frequency 0.022) with duplication of the entire *SOX9* gene and the upstream region spanning 13.5 kb, which may potentially contain regulatory elements. Such duplication was earlier reported in two out of seven XX DSD dogs (frequency 0.280) by Rossi et al [4], who used array-CGH. The duplication spanned 577 kb, however, and did not include the CNVR1 studied here. On the contrary, no *SOX9* duplication was identified by ddPCR in the *SOX9* coding region in XX DSD dogs from the model pedigree studied by Meyers-Wallen et al [8].

Further studies are needed to search for *SOX9* enhancers in dogs. We assume that 5’-flanking region of this gene may harbor crucial regulatory elements and that the CNV region located in the vicinity can lead to aberrant *SOX9* expression and the development of XX DSD phenotype. Variation of the canine XX DSD phenotype (testis or ovotestis, size and location of external genitalia, etc.) indicate that alterations in gene expression are probably responsible for this disorder, while loss-of-function gene mutations would seem to be unlikely. It should be pointed out that canine XX DSD affects only the reproductive system, and no cases were identified with skeletal dysplasia, which has been observed in some human syndromes with *SOX9* mutations, such as acampomelic campomelic dysplasia and Pierre Robin sequence [30]. This indicates that there are tissue-specific enhancers for testis development that are affected in XX DSD dogs. New technologies, such as ChIP-Seq and methods based on chromatin conformation capture, have recently been applied to human studies [23, 26] and should bring new data on cell-type regulation of *SOX9* gene expression also in dogs.

The distribution of G-insertion and cosegregating SNPs did not differ in the DSD and control cohorts. These results are opposite to those presented by Meyers-Wallen et al [8], where the incidence of heterozygotes (del/ins) in the DSD group was significantly higher than in the controls (Table E in S1 File). It is known that this polymorphism is located in intron 1 of the *BTBD17* gene. This gene is not strictly related to sex determination, but it has recently been found to be related to embryonic lethality in German Shorthair Pointers [8]. It cannot be excluded that the G-insertion is located in a regulatory element for testis-specific genes, or is associated with a causative mutation for XX DSD that segregates in particular breeds only.

## Conclusions

For the first time, we studied three different polymorphisms in the *SOX9* region in a large multibreed XX (*SRY*-negative) DSD cohort. CNVR1, upstream of *SOX9*, was highly polymorphic in both the DSD and control groups. We found that affected dogs had significantly higher numbers of copies. However, the association between XX DSD phenotype and CNV polymorphism was not pronounced in all the studied breeds. We speculate that this region may harbor key regulatory elements and, if multiplicated, can trigger *SOX9* expression in genetic females (XX, and lack of *SRY*). Among the DSD cases studied here, only one had duplication of the *SOX9* gene, which indicates that this mutation is rare in dogs. We did not confirm the association between G-insertion and DSD phenotype in our cohort. Finally, our study indicates that polymorphisms of the *SOX9* region do not explain the molecular background of all XX DSD cases. Further studies are needed to verify the role of the different mechanisms responsible for the regulation of *SOX9* (such as chromatin modifications and conformation), and other genomic regions that might be involved in the pathogenesis of this disorder should be taken into consideration.

## Supporting information

S1 File Supporting information (Tables and Figures). (PDF)
