# Supplementary material for: Association between polymorphisms in the SOX9 region and canine disorder of sex development (78,XX; SRY-negative) revisited in a multibreed case-control study
Source: PLoS One. 2019 Jun 20;14(6):e0218565. doi: 10.1371/journal.pone.0218565 (PMC6586338; doi:10.1371/journal.pone.0218565)
Supplement: S1 File — (PDF) [file pone.0218565.s001.pdf]

S1 File

Supporting information

Association between polymorphisms in the *SOX9* region and canine disorder of sex development (78,XX; *SRY*-negative) revisited in a multibreed case-control study

Short title: *SOX9* region polymorphisms in XX DSD dogs

Joanna Nowacka-Woszuk, Izabela Szczerbal, Monika Stachowiak, Maciej Szydlowski, Wojciech Nizanski, Stanislaw Dzimira, Artur Maslak, Rita Payan-Carreira, Eline Wydooghe, Tomasz Nowak, Marek Switonski*

* Correspondence to: Marek Switonski, switonsk@up.poznan.pl

Table A. Characteristics of the studied dogs

No	Breed	Gonadal status	Reference
DSD-1	French Bulldog	testis	[1]
DSD-2	French Bulldog	testis	[2]
DSD-3	French Bulldog	testis	[3]
DSD-4	French Bulldog	testis	this study
DSD-5	French Bulldog	ovotestis	
DSD-6	French Bulldog	testis	
DSD-7	French Bulldog	testis	
DSD-8	French Bulldog	testis	
DSD-9	French Bulldog	testis + ovotestis	
DSD-10	French Bulldog	testis	
DSD-11	French Bulldog	testis + ovary with tumor	
DSD-12*	French Bulldog	testis	
DSD-13	American Staffordshire Terrier	testis	[4]
DSD-14	American Staffordshire Terrier	NA	
DSD-15	American Staffordshire Terrier	NA	[2]
DSD-16	American Staffordshire Terrier	testis	[5]
DSD-17	American Staffordshire Terrier	ovotestis	this study
DSD-18	American Staffordshire Terrier	testis	
DSD-19	Cocker Spaniel	ovotestis	[6]
DSD-20	Cocker Spaniel	testis	[2]
DSD-21	Cocker Spaniel	NA	this study
DSD-22	German Shepherd	NA	[7]

DSD-23	German Shepherd	NA	
DSD-24	Yorkshire Terrier	NA	[2]
DSD-25	Yorkshire Terrier	ovotestis	this study
DSD-26	Pug	ovotestis	
DSD-27**	Pug	NA	[2]
DSD-28**	Pug	testis	
DSD-29***	Cane Corso	ovotestis	this study
DSD-30	Tibetan Terrier	NA	[5]
DSD-31	Bernese Mountain Dog	testis + ovotestis	[8]
DSD-32	Kerry Blue Terrier	NA	[5]
DSD-33	Beagle	ovotestis	[2]
DSD-34	Pyrenean Mountain Dog	NA	this study
DSD-35	Pointer	ovotestis	
DSD-36	Polish Lowland Sheepdog	NA	
DSD-37	Mongrel	testis	
DSD-38	Hollandse Smoushond	ovotestis	
DSD-39	American Bully	ovotestis	
DSD-40	Miniature Pinscher	NA	[4]
DSD-41	German Pinscher	NA	this study
DSD-42	Basenji	NA	
DSD-43	Pibull Terrier	testis	
DSD-44	Jack Russel Terrier	NA	
DSD-45	Austrian Pinscher	ovotestis	
C1-C8	French Bulldog (n = 8)	NA	[2] and this study
C9-C12	American Staffordshire Terrier (n = 4)	NA	[2] and this study
C13-C21	Cocker Spaniel (n = 9)	NA	[2] and this study

C22-C31	German Shepherd (n = 10)	NA	this study
C32-C45	Yorkshire Terrier (n = 14)	NA	this study
C46-C49	Pug (n = 4)	NA	[2] and this study
C50-C54	Beagle (n = 5)	NA	[2] and this study
C55-C56	Pointer (n = 2)	NA	this study
C57	Mongrel (n = 1)	NA	this study

*: DSD case from family A; **: DSD cases from family B; ***: DSD case from family C; NA: not analyzed

Table B. Four DSD cases and their family members

Family	Breed	DSD case	Healthy family members
A	French Bulldog	DSD-12	A1-father<br>A2-mother
B*	Pug	DSD-27<br>DSD-28	B1-father<br>B2-mother<br>B3-grandfather<br>B4-maternal grandmother<br>B5-paternal grandmother
C	Cane Corso	DSD-29	C1-father<br>C2-mother<br>C3-sister

*Two generations of this family were previously described by Marcinkowska-Swojak et al [2].

Table C. Characteristics of the assays used in ddPCR.

Assays	CanFam 3.1 location	Primers and probes	Reference
**CNVR1**			
CFA9_SOX9-CNV	Chr9 7876047-7876143	F:5’ TCATGCTTTTAATTATGACACA<br>R:5’ TAGTAACACAGACATTCCCT<br>probe:5’ FAM-TGACATATGTGTGCACGT-BQH1	this study
***SOX9* gene**			
CFA9_SOX9-5’-flanking	Chr9	BIO-RAD Assay ID: dCNS767522299	this study
CFA9_SOX9-exon 1	Chr9	BIO-RAD Assay ID: dCNS644292214	this study
CFA9_SOX9-exon 2	Chr9 8276496-8276650	F:5’ CAAGAAAGACCACCCGGATTACAA<br>R:5’ CGGAGGAGGAGTGCGGCGAGT<br>probe:5’ FAM-AGGAGGCCACCGAACAGACGCA-BQH1	[9]
CFA9_SOX9-exon 3	Chr9	BIO-RAD Assay ID: dCNS719223989	this study
**Reference gene**			
CFA38-HSD17B7	Chr38 19997088-19997185	F:5’ ATGTCCACACAACTAGCCATAC<br>R:5’ GTGTCTCGGTAGCGCATTT<br>probe:5’ HEX-CACGCCCAGTCCTAGTCATGCTT-BQH1	[9]

Table D. All identified genotypes for the three studied polymorphisms.

Groups	Animal ID	Genotypes after Sanger sequencing					Genotypes after ddPCR	
		rs852549625	rs22666734	rs9120764	rs851784236	rs852192717	Number of copies in upstream CNV	*SOX9* gene copies (exon 2)
DSD group	DSD-1	del/del	CC	GG	CC	GA	3	2
	DSD-2	del/del	CC	GG	CC	GA	3	2
	DSD-3	del/del	CC	GG	CC	GG	4	2
	DSD-4	del/del	CC	GG	CC	GA	0	2
	DSD-5	del/del	CC	GG	CC	GA	2	2
	DSD-6	del/del	CC	GG	CC	GA	2	2
	DSD-7	del/del	CC	GG	CC	GG	3	2
	DSD-8	del/del	CC	GG	CC	GG	2	2
	DSD-9	del/del	CC	GG	CC	GG	2	2
	DSD-10	del/del	CC	GG	CC	GA	2	2
	DSD-11	del/del	CC	GG	CC	GA	3	2
	DSD-12*	del/del	CC	GG	CC	AA	0	2
	DSD-13	del/del	CC	GG	CC	GG	3	2
	DSD-14	del/del	CC	GG	CC	GG	6	2
	DSD-15	del/del	CC	GG	CC	GG	6	2
	DSD-16	del/del	CC	GG	CC	GG	0	2
	DSD-17	del/del	CC	GG	CC	GG	1	2
	DSD-18	del/ins	CT	GA	CT	GG	2	2
	DSD-19	ins/ins	TT	AA	TT	GG	2	2
	DSD-20	del/del	CC	GG	CC	GG	6	2
	DSD-21	del/ins	CT	GA	CT	GG	0	2
	DSD-22	del/ins	CT	AA	CT	GG	2	2
	DSD-23	del/del	CC	GA	CC	GG	2	2
	DSD-24	del/del	CC	GG	CC	GG	5	2
	DSD-25	del/del	CC	GG	CC	GG	3	2
	DSD-26	del/del	CC	GG	CC	GA	2	2
	DSD-27**	ins/ins	TT	AA	TT	GG	7	2
	DSD-28**	ins/ins	TT	AA	TT	GG	6	2
	DSD-29***	del/ins	CT	GA	CT	GG	1	2
	DSD-30	del/ins	CT	GA	CT	GG	3	2
	DSD-31	del/del	CC	GG	CC	GG	5	2
	DSD-32	del/ins	CT	GA	CT	GG	4	2
	DSD-33	del/del	CC	GA	CC	GG	2	2
	DSD-34	del/del	CC	GG	CC	GG	0	2
	DSD-35	del/del	CC	GG	CC	GG	2	2
	DSD-36	del/del	CC	GG	CC	GG	0	2
	DSD-37	del/ins	CT	GA	CT	GG	5	2

	DSD-38	del/ins	CT	GA	CT	GG	3	2
	DSD-39	del/del	CC	GG	CC	GG	4	3
	DSD-40	del/del	CC	GG	CC	GG	4	2
	DSD-41	del/ins	CT	GA	CT	GG	3	2
	DSD-42	ins/ins	TT	AA	TT	GG	1	2
	DSD-43	del/del	CC	GG	CC	GG	3	2
	DSD-44	del/del	CC	GG	CC	GG	1	2
	DSD-45	del/del	CC	GG	CC	GG	2	2
Control group	C-1	del/del	CC	GG	CC	GA	0	2
	C-2	del/del	CC	GG	CC	GA	0	2
	C-3	del/del	CC	GG	CC	GG	3	2
	C-4	del/del	CC	GG	CC	GA	4	2
	C-5	del/del	CC	GG	CC	GA	0	2
	C-6	del/del	CC	GG	CC	GG	3	2
	C-7	del/del	CC	GG	CC	GG	3	2
	C-8	del/del	CC	GG	CC	GG	0	2
	C-9	del/del	CC	GG	CC	GG	1	2
	C-10	del/del	CC	GG	CC	GG	3	2
	C-11	del/del	CC	GG	CC	GG	2	2
	C-12	del/ins	CT	GA	CT	GG	4	2
	C-13	del/del	CC	GG	CC	GG	3	2
	C-14	del/del	CC	GG	CC	GG	1	2
	C-15	del/ins	CT	GA	CT	GG	1	2
	C-16	del/del	CC	GG	CC	GG	7	2
	C-17	del/ins	CT	GA	CT	GG	2	2
	C-18	del/del	CC	GG	CC	GG	2	2
	C-19	del/del	CC	GG	CC	GG	1	2
	C-20	del/ins	CT	GA	CT	GG	6	2
	C-21	del/del	CC	GG	CC	GG	6	2
	C-22	del/ins	CT	AA	CT	GG	1	2
	C-23	del/del	CC	GG	CC	GG	2	2
	C-24	del/ins	CT	AA	CT	GG	2	2
	C-25	del/del	CC	GA	CC	GG	1	2
	C-26	del/del	CC	GG	CC	GG	0	2
	C-27	del/del	CC	GG	CC	AA	0	2
	C-28	del/ins	CC	GA	CC	GG	0	2
	C-29	del/ins	CT	AA	CT	GG	1	2
	C-30	ins/ins	TT	AA	TT	GG	1	2
	C-31	del/ins	CT	GA	CT	GG	0	2
	C-32	del/del	CC	GG	CC	GG	2	2
	C-33	del/del	CC	GG	CC	GG	1	2
	C-34	del/del	CC	GG	CC	GG	4	2
	C-35	del/del	CC	GG	CC	GG	0	2
	C-36	del/del	CC	GG	CC	GG	0	2
	C-37	del/del	CC	GG	CC	GG	1	2

	C-38	del/del	CC	GG	CC	GG	4	2
	C-39	del/del	CC	AA	CC	GG	2	2
	C-40	del/del	CC	GG	CC	GG	1	2
	C-41	del/del	CC	GG	CC	GG	6	2
	C-42	del/del	CC	GG	CC	GG	0	2
	C-43	del/del	CC	GG	CC	GG	2	2
	C-44	del/ins	CT	GA	CT	GG	2	2
	C-45	del/del	CC	GG	CC	GG	1	2
	C-46	del/del	CC	GG	CC	GG	0	2
	C-47	del/ins	CT	GA	CT	GG	2	2
	C-48	del/del	CC	GG	CC	GG	5	2
	C-49	del/del	CC	GG	CC	GG	0	2
	C-50	del/del	CC	GG	CC	GG	1	2
	C-51	del/del	CC	GG	CC	GG	3	2
	C-52	del/del	CC	GG	CC	GG	1	2
	C-53	del/del	CC	GG	CC	GG	6	2
	C-54	del/del	CC	GA	CC	GG	3	2
	C-55	del/ins	CT	AA	CT	GG	2	2
	C-56	del/ins	CT	GA	CT	GG	2	2
	C-57	del/del	CC	GG	CC	GG	3	2
Family A members	A1-father	del/del	CC	GG	CC	GA	0	2
	A2-mother	del/del	CC	GG	CC	GA	3	2
Family B members	B1-father	ins/ins	TT	AA	TT	GG	5	2
	B2-mother	del/ins	CT	GA	CT	GG	2	2
	B3-grandfather	del/ins	CT	GA	CT	GG	3	2
	B4-maternal grandmother	del/del	CC	GG	CC	GG	0	2
	B5-paternal grandmother	del/ins	CT	GA	CT	GG	3	2
Family C members	C1-father	del/ins	CC	GG	CC	GG	0	2
	C2-mother	ins/ins	TT	AA	TT	GG	2	2
	C3-sister	del/ins	CT	GA	CT	GG	1	2

*: DSD case from family A; **: DSD cases from family B; ***: DSD case from family C; NA: not analyzed

Table E. Genotype frequencies of the G-insertion (rs852549625) in DSD and control groups. The results are presented in comparison to those reported by Meyers-Wallen et al [9].

Groups	Genotype frequencies		
	**del/del**	**del/ins**	**ins/ins**
DSD (n = 44)* - this study	0.70	0.20	0.10
Control (n = 57) – this study	0.75	0.23	0.02
DSD (n = 61) – based on Meyers-Wallen et al [9]	0.21	0.76	0.03
control 1 (n = 64) – based on Meyers-Wallen et al [9]	0.49	0.42	0.09
control 2 (n = 57) – based on Meyers-Wallen et al [9]	0.69	0.26	0.05
control 1+2 (n = 121) – based on Meyers-Wallen et al [9]	0.58	0.35	0.07

* DSD-39 was not included in this analysis, as it was a carrier of *SOX9* gene duplication

Table F. Haplotypes frequencies in DSD and control groups.

Groups	Haplotype				
	**DelCGCG**	**InsTATG**	**DelCGCA**	**DelCACG**	**InsCACG**
DSD all cases (n = 44)*	0.659	0.193	0.114	0.034	-
control all (n = 57)	0.746	0.123	0.070	0.053	0.008
DSD French Bulldog (n = 12)	0.625	-	0.375	-	-
control French Bulldog (n = 8)	0.750	-	0.250	-	-
DSD without French Bulldog (n = 32)	0.672	0.266	0.015	0.047	-
control without French Bulldog (n = 49)	0.745	0.143	0.020	0.082	0.010
DSD American Staffordshire Terrier (n = 6)	0.917	0.083	-	-	-
control American Staffordshire Terrier (n = 4)	0.875	0.125	-	-	-
DSD without American Staffordshire Terrier (n = 38)	0.618	0.211	0.132	0.039	-
control without American Staffordshire Terrier (n = 53)	0.736	0.123	0.057	0.075	0.009
DSD without French Bulldog and American Staffordshire Terrier (n = 26)	0.615	0.308	0.019	0.058	-
control without French Bulldog and American Staffordshire Terrier (n = 45)	0.733	0.144	0.022	0.089	0.012

Order in haplotypes: G-insertion (rs852549625) and 4 SNPs (rs22666734; rs9120764; rs851784236; rs852192717)

* DSD-39 was excluded from this analysis, as it was a carrier of *SOX9* gene duplication

Table G. Canine breeds found to suffer from XX DSD. The first row shows all breeds reported to 2012 and reviewed by Meyers-Wallen et al [10]; the following rows list newer breeds.

Breed	Reference
American Cocker Spaniel, Afghan Hound, American Pit Bull Terrier, American Staffordshire Terrier, Australian Shepherd, Basset hound, Bernese Mountain Dog, Beagle, Border Collie, Brussels Griffon, Doberman Pinscher, English Cocker Spaniel, French Bulldog, German Pinscher, German Shepherd Dog, German Shorthaired Pointer, Golden Retriever, Jack Russell Terrier, Kerry Blue Terrier, Mixed Breed, Norwegian Elkhound, Podenco Dog, Pug, Soft Coated Wheaten Terrier, Tibetan Terrier, Vizsla, Walker Hound, Weimaraner, Wheaten Terrier	Reviewed by Meyers-Wallen et al [10]
Leonberger, Miniature Pinscher, Yorkshire Terrier	[5]
Pit Bull Terrier	[9]
American Bully, Basenji, Cane Corso, Hollandse Smoushond, Polish Lowland Sheepdog, Pyrenean Mountain Dog	This study

Figure A. Pedigrees of the three studied families. Family A: French Bulldog; Family B: Pug; Family C: Cane Corso. Details of animal numbering are shown in S1 and S2 Tables.

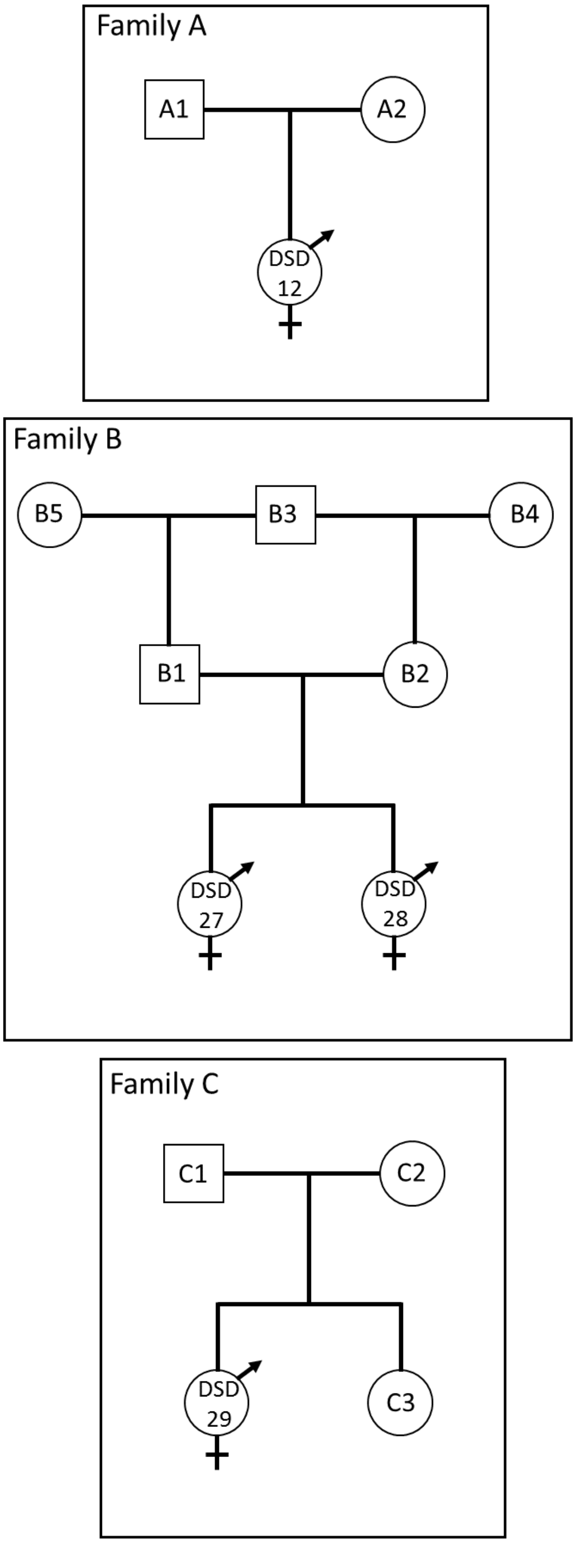

Figure B. Diagnosis of a new DSD case (French Bulldog, DSD-4). An enlarged clitoris (a); Giemsa stained metaphase spread – two X chromosomes are indicated (b); detection of *SRY* gene by PCR and electrophoresis (amplicon of 813 bp) – DSD-4 analyzed in duplicate, M1-M2 – control males, F1-F2 – control females, 0 – negative control with no DNA template (c); histological analysis of gonads – presence of inactive testes (d).

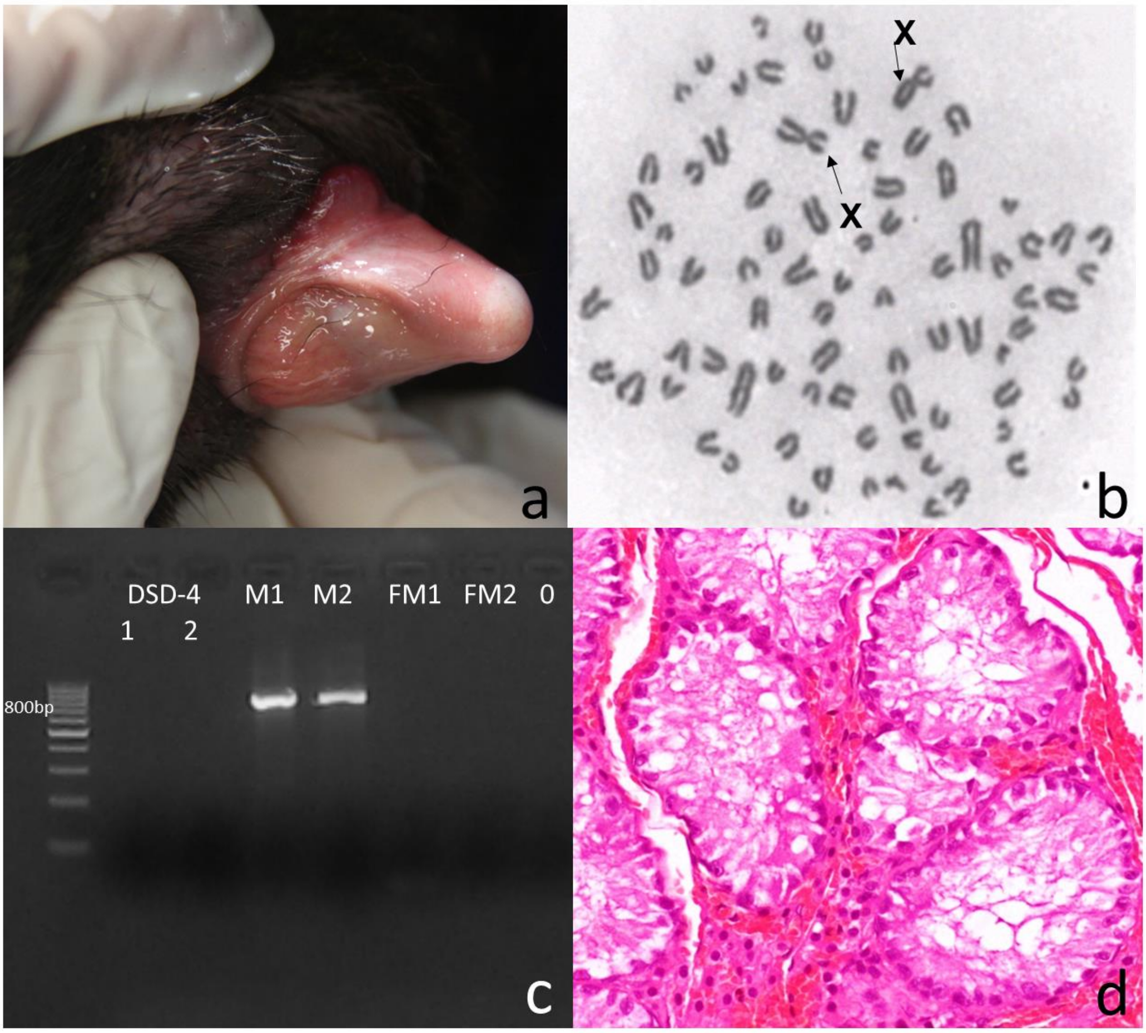

Figure C. CNVR1 polymorphism studied by ddPCR in the case and control groups. Variability in copy number from 0 to 7 in DSD (a) and from 0 to 6 in the controls is shown (b). nk: negative control (no DNA). Error bars represent the 95% confidence interval.

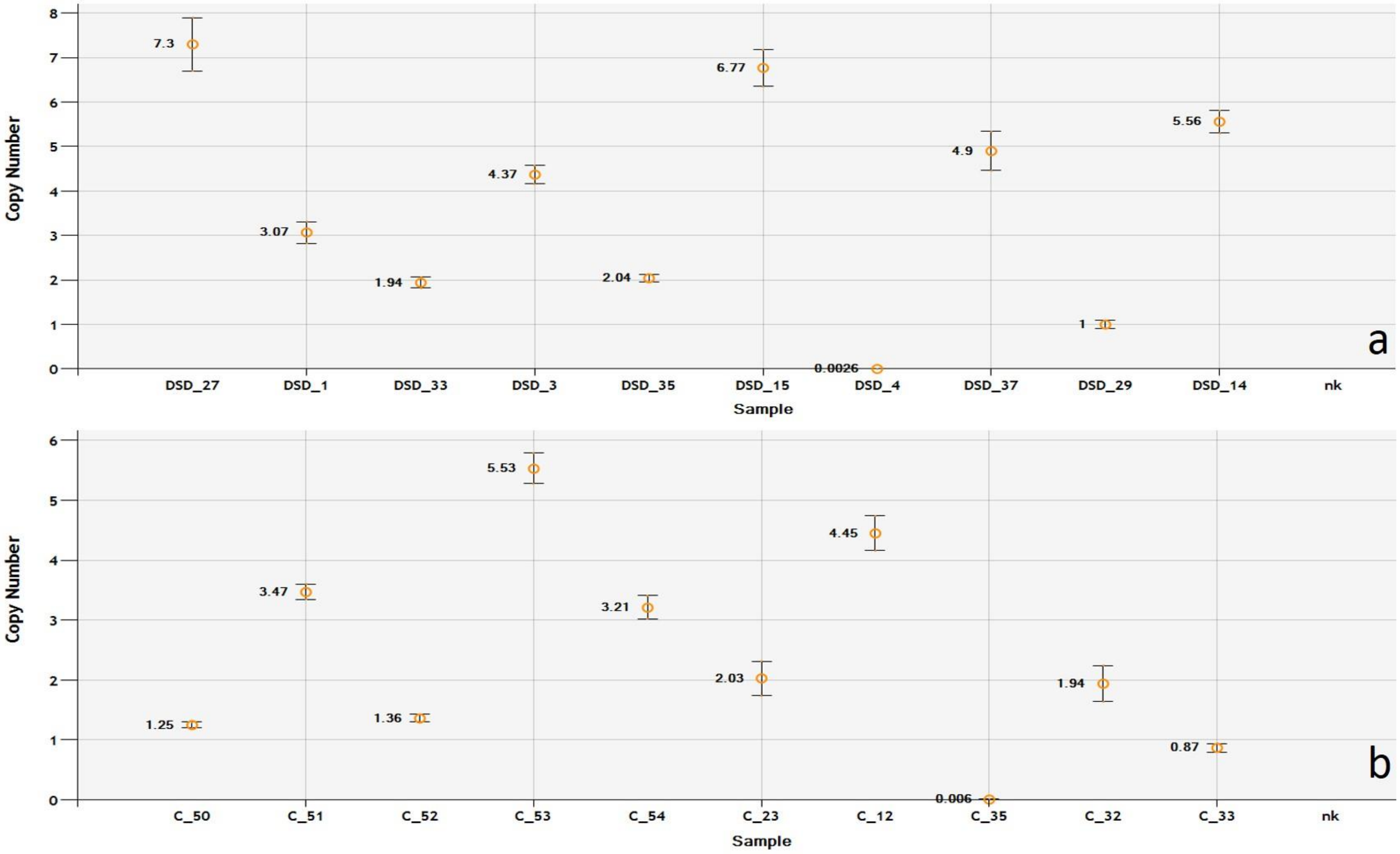

Figure D. Segregation of CNVR1 polymorphism in the Pug family. The red FISH signals is specific to CNVR1, while the green signal was used for the control probe (*SOX9* gene). Individuals B1, B2, DSD-27, and DSD-28 have previously been studied by Marcinkowska-Swojak et al [2]. The explanation of animal's numbering is given in S2 Table. Genotypes for G-insertion are shown for each individual.

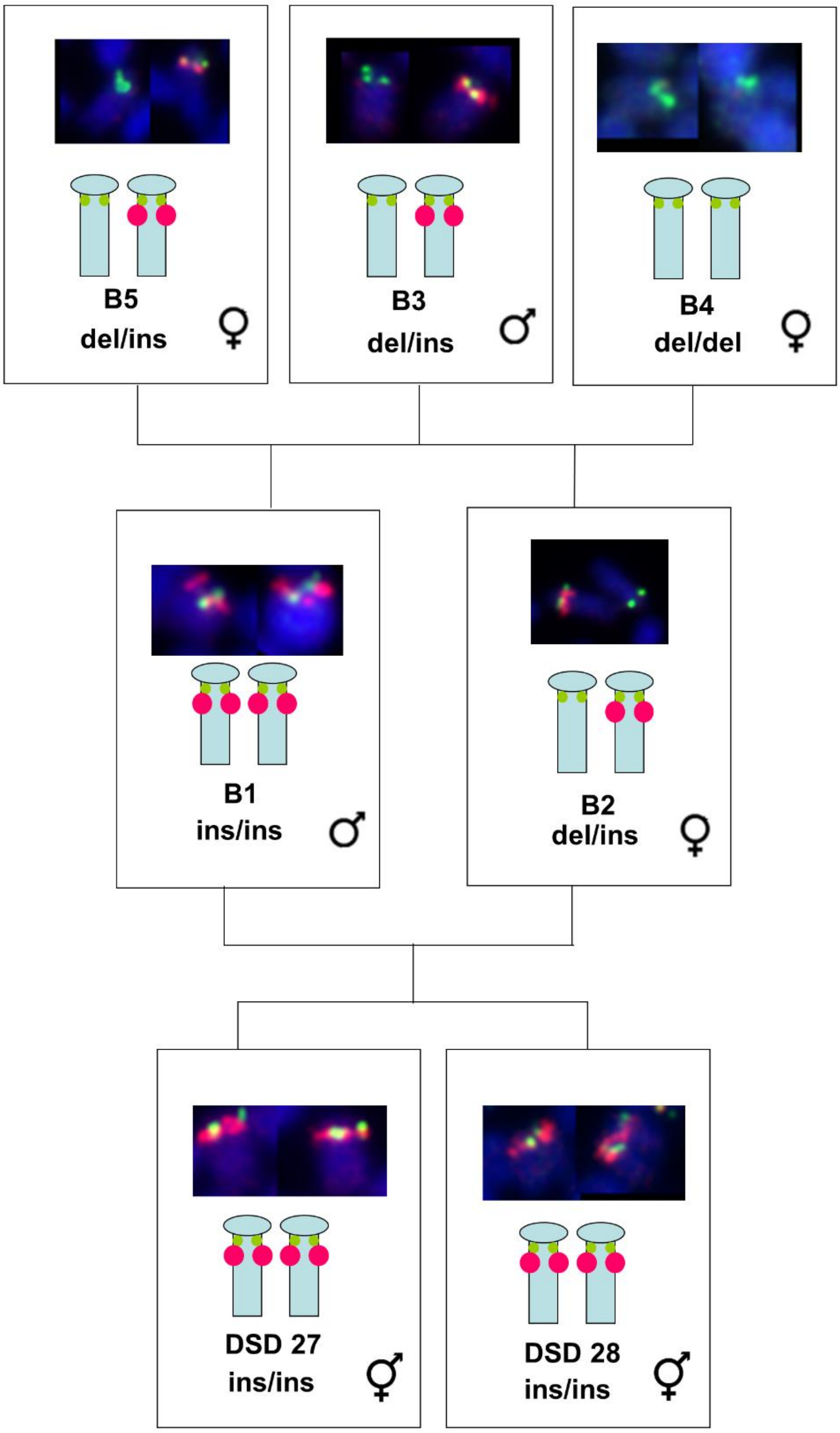

Figure E. The segregation of CNVR1 polymorphism in the Cane Corso family. Analysis was performed using interphase FISH (I-FISH). The red FISH signals is specific to CNVR1, while the green signal was used for the control probe (*SOX9* gene). Co-localisation of both probes on chromosome 9 territory is marked by ellipse. The explanation of animal's numbering is given in S2 Table. Genotypes for G-insertion are shown for each individual. Note that the BAC probe specific to CNVR1 also hybridized to chromosome 18 territory due to a homologous fragment.

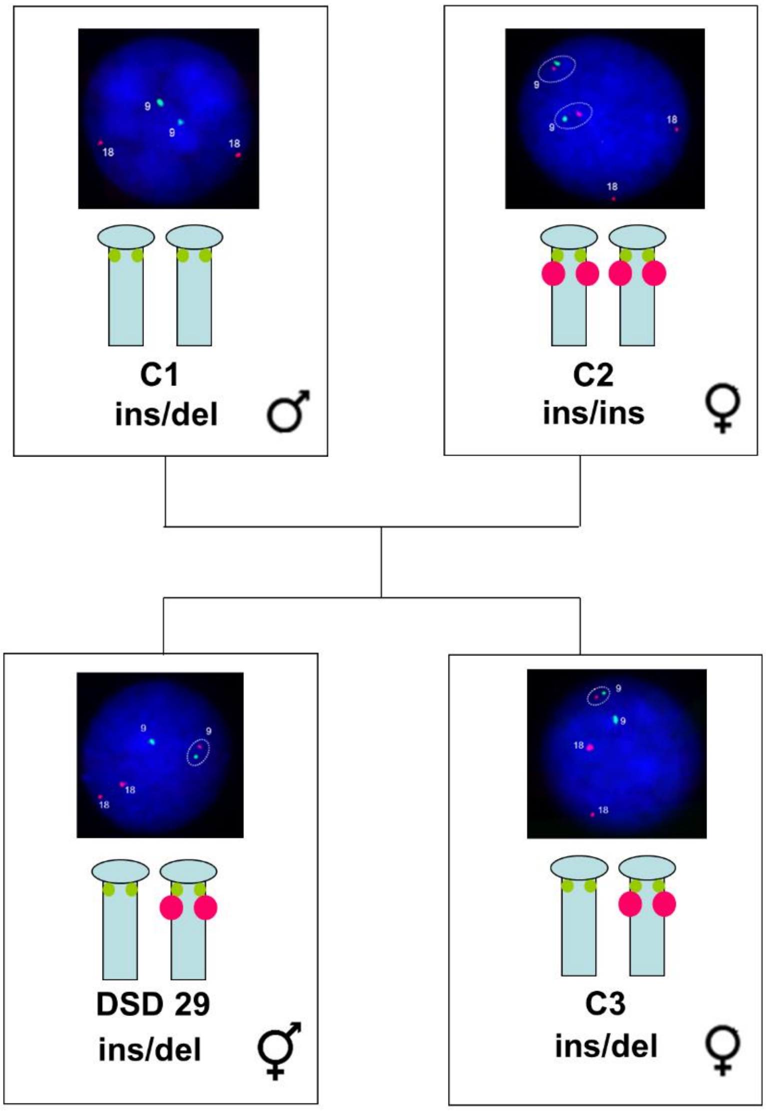

Figure F. The segregation of CNVR1 polymorphism in the French Bulldog family. The red FISH signals is specific to CNVR1, while the green signal was used for the control probe (*SOX9* gene). The explanation of animal's numbering is given in S2 Table. Genotypes for G-insertion are shown for each individual.

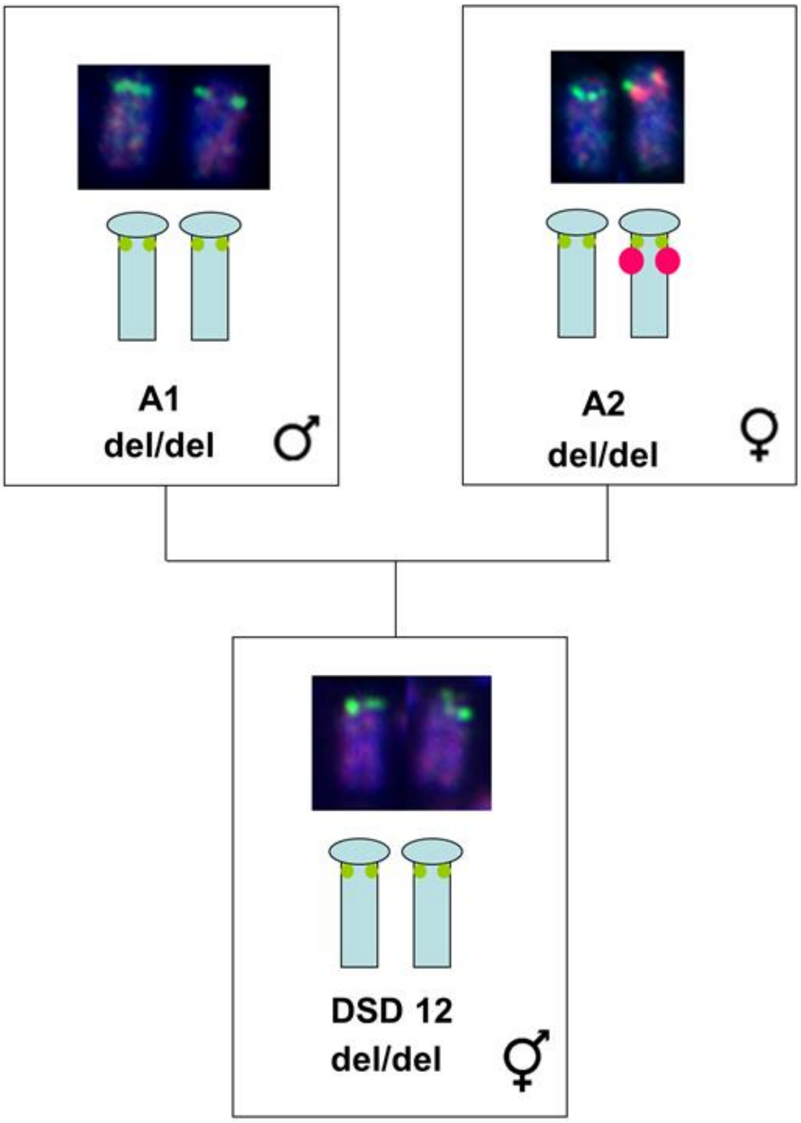

References to supporting information

1. Szczerbal I, Nowacka-Woszuk J, Dzimira S, Atamaniuk W, Nizanski W, Switonski M. A Rare Case of Testicular Disorder of Sex Development in a Dog (78, XX; SRY-Negative) with Male External Genitalia and Detection of Copy Number Variation in the Region Upstream of the SOX9 Gene. Sexual Development. 2016;10(2):74-8. doi: 10.1159/000445464. PubMed PMID: WOS:000376570900003.

2. Marcinkowska-Swojak M, Szczerbal I, Pausch H, Nowacka-Woszuk J, Flisikowski K, Dzimira S, et al. Copy number variation in the region harboring SOX9 gene in dogs with testicular/ovotesticular disorder of sex development (78,XX; SRY-negative). Sci Rep-Uk. 2015;5. doi: ARTN 14696 10.1038/srep14696. PubMed PMID: WOS:000362094000001.

3. Van Cleven A, Wydooghe E, Van Brantegem L, Szczerbal I, Stachowiak M, Switonski M, et al. Testicular disorder of sex development (78,XX SRY-negative) in a female French bulldog. Vlaams Diergen Tijds. 2015;84(6):318-25. PubMed PMID: WOS:000367501000003.

4. Nowacka J, Nizanski W, Klimowicz M, Dzimira S, Switonski M. Lack of the SOX9 gene polymorphism in sex reversal dogs (78,XX; SRY negative). Journal of Heredity. 2005;96(7):797-802. doi: 10.1093/jhered/esi106. PubMed PMID: WOS:000233492400014.

5. Salamon S, Nowacka-Woszuk J, Szczerbal I, Dzimira S, Nizanski W, Ochota M, et al. A Lack of Association between Polymorphisms of Three Positional Candidate Genes (CLASP2, UBP1, and FBXL2) and Canine Disorder of Sexual Development (78,XX; SRY-Negative). Sexual Development. 2014;8(4):160-5. doi: 10.1159/000363531. PubMed PMID: WOS:000340346500005.

6. Switonski M, Payan-Carreira R, Bartz M, Nowacka-Woszuk J, Szczerbal I, Colaco B, et al. Hypospadias in a Male (78,XY; SRY-Positive) Dog and Sex Reversal Female (78,XX; SRY-Negative) Dogs: Clinical, Histological and Genetic Studies. Sexual Development. 2012;6(1-3):128-34. doi: 10.1159/000330921. PubMed PMID: WOS:000300516900013.

7. Switonski M, Nowacka J, Skorczyk A, Chmurzyńska A, Niżański W. Dziedziczny zespół odwróconej płci (78,XX; brak genu SRY) u szczeniąt owczarka niemieckiego. Medycyna Weterynaryjna. 2004;60(7):705-7.

8. Switonski M, Szczerbal I, Nizanski W, Kociucka B, Bartz M, Dzimira S, et al. Robertsonian Translocation in a Sex Reversal Dog (XX, SRY negative) May Indicate that the Causative Mutation for This Intersexuality Syndrome Resides on Canine Chromosome 23 (CFA23). Sexual Development. 2011;5(3):141-6. doi: 10.1159/000324689. PubMed PMID: WOS:000291350600005.

9. Meyers-Wallen VN, Boyko AR, Danko CG, Grenier JK, Mezey JG, Hayward JJ, et al. XX Disorder of Sex Development is associated with an insertion on chromosome 9 and downregulation of RSPO1 in dogs (Canis lupus familiaris). Plos One. 2017;12(10). doi: ARTN e0186331 10.1371/journal.pone.0186331. PubMed PMID: WOS:000413315100017.

10. Meyers-Wallen VN. Gonadal and Sex Differentiation Abnormalities of Dogs and Cats. Sexual Development. 2012;6(1-3):46-60. doi: 10.1159/000332740. PubMed PMID: WOS:000300516900005.
